# Extracellular vesicles derived from EphB2-overexpressing bone marrow mesenchymal stem cells ameliorate DSS-induced colitis by modulating immune balance

**DOI:** 10.1186/s13287-021-02232-w

**Published:** 2021-03-15

**Authors:** Ting Yu, Si Chu, Xingxing Liu, Junyi Li, Qianyun Chen, Meng Xu, Hui Wu, Mingyue Li, Yalan Dong, Feng Zhu, Haifeng Zhou, Desheng Hu, Heng Fan

**Affiliations:** 1 Department of Integrated Traditional Chinese and Western Medicine, Union Hospital, Tongji Medical College, Huazhong University of Science and Technology, Wuhan, 430022 China; 2 Department of Hematology, Union Hospital, Tongji Medical College, Huazhong University of Science and Technology, Wuhan, 430022 China

**Keywords:** Extracellular vesicles, Bone marrow mesenchymal stem cells, Ulcerative colitis, EphB2, Homing, Th17/Treg balance

## Abstract

**Background:**

The bone marrow mesenchymal stem cell (BMSCs)-derived extracellular vesicles (EVs) open up a new avenue for ulcerative colitis (UC) treatment recently, but they are not selectively enriched in targeted tissues. EphB2, a cell-to-cell signaling receptor, is identified as a regulator for inflammatory response, immune homeostasis and cell migration. In this study, we investigated the therapeutic potential and underlying mechanism for EphB2 over-expressing BMSCs derived EVs (EphB2-EVs) in the treatment of UC.

**Methods:**

BMSCs and EVs were obtained and characterized by a series of experiments. Lentivirus vector encoding EphB2 was transfected into BMSCs and verified by qRT-PCR. We analyzed the EphB2-EVs ability of colonic targeting in a DSS-induced colitis model by using confocal microscope and WB. The protective effect of EphB2-EVs in vivo was systematically evaluated by using a series of function experiments.

**Results:**

We successfully constructed EphB2-overexpressing BMSCs derived EVs (EphB2-EVs). Overexpression of EphB2 significantly enhanced the homing of EVs to the damaged colon. In addition, EphB2-EVs were effective to attenuate inflammation in intestinal mucosa and restore the damaged colon tissue by inhibiting the release of proinflammatory cytokines and upregulating the anti-inflammatory mediators. EphB2-EVs effectively reduced the oxidative stress and repaired the intestinal mucosal barrier in the UC rats. Moreover, EphB2-EVs demonstrated a robust immunomodulatory effect to restore immune homeostasis via modulating Th17/Treg balance and restraining STAT3 activation.

**Conclusions:**

Our results suggest that EphB2-EVs have high colonic targeting ability and could mitigate DSS-induced colitis via maintaining colonic immune homeostasis. These findings provide an effective therapeutic strategy for UC treatment in clinic.

## Background

Ulcerative colitis (UC) is a relapsing and refractory inflammatory bowel disease and has gradually becoming a health challenge worldwide [1]. It greatly affects patient’s life quality due to the bloody diarrhea, mucus discharge, abdominal pain, and fecal urgency [2–4]. Current treatment for UC showed dubious curative effect and potential side effects. Therefore, the safer and more effective therapeutic approaches for UC are constantly being sought.

Despite decades of research, the etiology of UC remains elusive. It has been suggested that gene susceptibility, damaged intestinal mucosal and epithelial barrier, alteration of intestinal flora, disturbed immune homeostasis, and environmental factors are involved in the pathogenesis of UC [5–7]. Among which, the aberrant innate and adaptive immunity plays a key role in UC pathogenesis, especially T cells in maintaining intestinal immune homoeostasis [8]. Though Th2-mediated immune response was initially regarded as a driven force of UC, emerging evidence indicates that UC is caused by excessive activation of Th17 cells or deficiency of Treg cells [9–11]. Hence, modulating the balance of Th17/Treg cells might be an effective therapeutic strategy for UC.

Erythropoietin-producing hepatocellular (Eph) receptors comprise the largest family members of receptor tyrosine kinases (RTKs) whose cognate ligands are the ephrins [12, 13]. Eph receptor interacts with its ligand in a bidirectional manner [14]. Both EphB2 and ephrin-B1 are cell surface proteins, and the former can normally be activated by cell to cell contact [15]. The activation of EphB2/ephrin-B1 reverse signaling can exert a wide range of biological effects [16–19]. Notably, the EphB2/ephrin-B1 signaling has shown to be engaged in the differentiation and function of immune cells. Activation of EphB2/ephrin-B1 axis results in T cell inhibition [20]. And the ephrin-B1 reverse signaling inhibits T cell proliferation [21]. In addition, over-expressed ephrin-B1 in inflammatory conditions has high affinity to its cognate receptors, rendering the EphB2/ephrin-B1 axis crucial in cell migration to the inflamed tissue [22]. These studies demonstrated the involvement of EphB2/ephrin-B1 axis in inflammatory diseases.

In recent years, the bone marrow mesenchymal stem cells (BMSCs) have shown to be efficacious to UC, likely through suppressing inflammatory response and regulating immune homeostasis [23–26]. The therapeutic effect of BMSCs is attributed to their paracrine action which is mainly mediated by extracellular vesicles (EVs) [27, 28]. EVs (30 nm–2000 nm diameter) mediate cell-cell communication through lipid, nucleic acid, and protein cargoes [29, 30] and are thus involved in a great variety of physiological or pathological processes, such as tissue repair, immune surveillance, and cell proliferation [31–34]. Due to their stability and low immunogenicity compared to their parent cells, EVs have been regarded as a novel therapeutic tool or carrier [27]. Indeed, previous studies have demonstrated that BMSC-derived EVs are beneficial to UC [35–37]. Meanwhile, as a promising mediator of intercellular communication and drug delivery carrier, EVs have been widely used in the research of various diseases [38, 39]. However, beside their low productivity, EVs are largely retained within the spleen, lung, and liver after systemic administration, which limit their accumulation in the targeted organs like colon [40, 41]. Intriguingly, previous studies have shown that EphB2-carried EVs could be transported for long-distance to exert biological effects [12, 19]. These findings lead to the hypothesis that EphB2 might provide a new approach to enhance the enrichment of EVs in the lesion area of UC, so as to increase their therapeutic efficacy.

In this study, we evaluated the role of EphB2/ephrin-B1 axis in the enrichment of EVs in the damaged colon and the effects of EphB2-EVs on DSS-induced intestinal inflammation. In addition, the underlying immunological mechanisms were explored. Our results showed that a large proportion of EphB2-EVs was localized in the damaged colon, and the EphB2-EVs robustly attenuated intestinal inflammation via modulating Th17/Treg balance.

## Materials and methods

### Animals

Male Sprague-Dawley (SD) rats, weighing 180–200 g, were purchased from the experimental animal center of Huazhong University of Science and Technology (HUST, Wuhan, China) and housed under specific-pathogen-free (SPF) conditions. This study was conducted strictly according to the Animal Research Institute Committee guidelines of HUST. All animal experiments procedures were approved by the Institutional Animal Care and Use Committee (IACUC) of HUST.

### Isolation, cultivation, and identification of rats BMSCs

BMSCs were isolated and cultured from the healthy male SD rats weighing 80–100 g as described previously [35]. Cells at 3rd to 5th passage (P3 to P5) were utilized for subsequent experiments. The morphology of the BMSCs was observed under a common optical microscope. The P3 BMSCs were identified by flow cytometry and the phenotype was characterized with the following antibodies: PE-Cy7-anti-CD29, Alexa Fluor 488-anti-CD90, Alexa Fluor 647-anti-CD11b, and PE-anti-CD45 (BioLegend, San Diego, CA, USA). BMSCs were used as the EVs donor cells in the study.

### Construction of recombinant lentivirus

The rat EphB2 gene fragments were acquired through a chemical synthesis method completed by Shanghai Generay Biotech Co., Ltd. The lentiviral vector, Ubi-MCS-3FLAG-CBh-gcGFP-IRES-puromycin (named GV492; Genechem, Shanghai, China), was digested by the restriction enzyme BamHI/AgeI. The EphB2 gene fragments were ligated into the lentiviral vector GV492 mentioned above. The primers (5′-GTCAACACGCTGGACAAGAT-3′ and 5′-CCTTATAGTCCTTATCATCGTC-3′) located in the vector were used in PCR to identify positive transformants. Positive clones, with length of 394 bp, were chosen for sequencing. Recombinant lentiviruses, which co-expressed gc green fluorescent protein (gcGFP), anti-puromycin gene, and EphB2, were produced by 293T cells following the cotransfection with GV492 and the packaging plasmids pHelper 1.0 and pHelper 2.0 (Genechem). The virus titer was detected through a drug screening method.

### In vitro transduction with lentivirus and culture expansion

When P2 BMSCs reached 20–40% confluence in the T25 culture flask, transfection was performed at a multiplicity of infection of 35 in the presence of 100 μl HitransG P (Genechem) following the manufacturer’s instructions. EphB2-BMSCs were genetically engineered with recombinant lentivirus expressing the EphB2, anti-puromycin gene, and gcGFP. And null-BMSCs were manipulated with lentivirus expressing the anti-puromycin gene and gcGFP which were used as a negative control. Successfully transfected cells were selected with puromycin at a final concentration of 2.5 mg/mL (Sigma-Aldrich, St. Louis, MO, USA) for 7 days. All these cells were expanded to P3 to P5 generation and used for the EVs harvest.

### Preparation and characterization of EVs

EVs were isolated from the supernatant of BMSCs by ultracentrifugation when the P3 to P5 BMSCs reached to 70–80% confluence (Figure S1). Briefly, BMSCs were thoroughly washed with phosphate-buffered saline (PBS) for three times and starved by serum-free low-glucose DMEM media for 48 h at 37 °C. Next, cell culture supernatants were collected followed by centrifugation at 300 g for 10 min to remove dead cells. Then, the supernatants were centrifuged at 2000×*g* for 25 min and 10,000×*g* for 30 min to eliminate cellular debris. The EVs were isolated from cell free supernatants by ultracentrifugation (Beckman Coulter OptimaL-100 K ultracentrifuge, Fullerton, CA, USA) at 110,000×*g* for 90 min at 4 °C, resuspended in sterile PBS and subjected to a second ultracentrifugation under the same conditions. The milk-white sediments in the bottom of the centrifuge tube were the purified EVs. The protein concentration of the EVs was quantified by BCA assay (Beyotime Biotechnology, Shanghai, China). The EVs were resuspended in PBS and stored at − 80 °C until use. The morphology of isolated EVs was observed under 200 kV transmission electron microscope (TEM) (Tecnai G^2^ 20 TWIN, FEI, USA). The size distribution was analyzed by Nanoparticle Tracking Analysis (NTA) (NanoSight, Malvern, UK). Surface epitope protein expression (CD9, CD63, and TSG101) in EVs were identified by Western blotting.

### Induction of experimental colitis and animal treatment

Rat model of acute experimental colitis was induced by 5.0% (wt/vol) DSS (molecular weight: 36–50 kDa; MP Biomedicals, Illkirch, France) added in their purified drinking water for 7 days, according to methods described by Ma et al. [42]. Briefly, forty rats were randomly assigned to 5 groups (*n* = 8): the normal group, the DSS group, the EVs group, the EphB2-EVs group, and the mesalazine group, and the rats were allowed to acclimatize for 1 week before the experiment procedure. After adaptive feeding, colitis was induced by 5% DSS for 1 week. After the modeling, rats in normal group were administered with purified drinking water orally; while DSS and treatment groups were continuously fed with 1% DSS for 1 week. On the 8th day, the animals in the EVs and EphB2-EVs groups received corresponding EVs suspended in 1 mL PBS via tail vein at a dose of 100 μg per rat. On the day 11, the same administration as described above was performed again in both groups. Mesalazine group as positive control received mesalazine (0.42 g/kg). The drugs were given orally once daily and continued for 1 week. On the day 15, all the rats were anesthetized and an aseptic laparotomy was performed. The colons, spleens, and mesenteric lymph nodes (MLNs) were obtained and stored for subsequent studies.

To investigate the location and distribution of BMSC-EVs in colon tissue after administration, another 6 colitis rats received the EVs and EphB2-EVs respectively which were labeled with PKH26 (Sigma-Aldrich), a red fluorescent dye, under the guidance of manufacturer as previously described [36]. The frozen sections of colon were observed by the laser scanning confocal microscope (Olympus-FV1000, Tokyo, Japan).

### Assessment of colitis

During DSS treatment, diet, body weights, stool consistency, and stool occult blood were daily observed to evaluate the disease activity index (DAI) of colitis in rats. The DAI scores are shown in Table S1, adapted from Yang et al. [35]. On the day 15, the length of the colon was measured. Then, colons were dissected, fixed and stained with hematoxylin and eosin (H&E) for histological analysis.

### mRNA qRT-PCR

The mRNA expressions of EphB2, ephrin-B1, ROR-γt, and Foxp3 were quantified by qRT-PCR as described previously [43]. The mRNAs expression was normalized to β-actin. All mRNA primers were listed in Table S2.

### Western blotting (WB)

Western blotting analysis of colon tissue was performed according to the previous description [35]. Anti-EphB2 (1:1000, Cell Signaling Technology, Danvers, MA, USA), anti-Eph receptor B1 (1:2000, Abcam, Cambridge, UK), anti-STAT3 (1:1000, Cell Signaling Technology), anti-pSTAT3 (Tyr705) (1:2000, Cell Signaling Technology), anti-NF-κBp65 (1:2000, Cell Signaling Technology), anti-pNF-κBp65 (Ser536) (1:1000, Cell Signaling Technology) antibodies were used as primary antibodies respectively. The protein expression was normalized to β-actin.

### Immunohistochemistry (IHC) and immunofluorescent (IF) staining

Immunohistochemistry and immunofluorescence staining were performed as previously described [44]. Anti-STAT3 (1:300, Cell Signaling Technology) antibody was used as primary antibodies for immunohistochemistry. For immunofluorescence, the tissue sections were placed in a box filled with EDTA antigen repair buffer (pH 9.0), then incubated overnight at 4 °C in a humidified environment with Anti-ZO-1 (Affiinity Cat# AF5145) and Anti-occludin (Abclonal Cat# A2601). Sections were then counterstained with DAPI, washed, and mounted with anti-fading medium. Images were collected on Nikon inverted fluorescence microscope. The location of PKH26 labeled EVs in the intestine were observed by a laser confocal scanning microscope (Olympus-FV1000, Tokyo, Japan).

### Enzyme-linked immunosorbent assay (ELISA)

The expression levels of TNF-α, IFN-γ, IL-1β, IL-2, IL-17A, IL-10, IL-6, and TGF-β1 in colon homogenate supernatants of rats were measured using ELISA kits (Bioswamp, Wuhan, China) according to the manufacturer’s instruction. The assays of these cytokines used the quantitative sandwich enzyme immunoassay technique and the absorbance was measured at 450 nm employing microplate reader.

### Flow cytometry

Mononuclear cells were isolated from spleen and mesenteric lymph nodes (MLNs) as described previously [45]. For Th17 cell staining, cells were incubated and restimulated using a leukocyte activation cocktail with BD GolgiPlug™ (BD Biosciences) at 37 °C, 5% CO_2_ for 9 h. Then, surface markers were stained with FITC-anti-CD45 antibody and PE-Cy7-anti-CD4 antibody (BD Biosciences, San Diego, CA, USA) at 4 °C for 30 min in dark. Subsequently, cells were fixed and permeabilized with fixation/permeabilization solution (BD Biosciences) for 20 min, then washed in BD Perm/Wash™ buffer (BD Biosciences), and next stained intracellularly with eFluor 450-anti-IL-17A antibody (eBioscience, San Diego, CA, USA) for 45 min. After washing, stained cells were analyzed by flow cytometry. For Treg cell staining, surface markers of which were stained with FITC-anti-CD45 antibody and PE-Cy7-anti-CD4 antibody (BD Biosciences) at 4 °C for 30 min in dark, then fixed and permeabilized with Fix/Perm Buffer working solution and Perm/Wash Buffer (BD Biosciences) at 4 °C for 45 min. After that, PE-anti-Foxp3 antibody (eBioscience, San Diego, CA, USA) was used for intranuclear staining for 30 min. Finally, the stained cells were analyzed by flow cytometry.

### Biochemical detection

Freshly excised colon was gently rinsed in PBS to remove feces, homogenized in tissue lysis buffer and centrifuged. The activities of myeloperoxidase (MPO), superoxide dismutase (SOD) and the concentration of glutathione (GSH), malondialdehyde (MDA) in the colon were detected using the commercial assay kit (Nanjing Jiancheng Bioengineering Institute, Nanjing, China) according to the manufacturer’s instruction.

### Transmission electron microscopy (TEM)

EVs and colon tissues were prepared by special methods to observe their morphological structure under TEM. EVs were handled as described previously [37]. For colon tissues, it was soaked in 2.5% glutaraldehyde and rinsed with 0.1 M phosphate buffer (pH 7.4) for 3 times, each time for 15 min. Samples were fixed with 1% osmic acid × 0.1 M phosphate buffer (pH 7.4) at room temperature for 2 h. Then, rinse with 0.1 M phosphate buffer (pH 7.2) for 3 times, each time for 15 min. After dehydration through a graded ethanol series, samples were added with penetrant. The prepared samples were embedded and sliced to 80–100 nm. After staining with 1% uranyl acetate and 0.4% lead citrate, samples were dried overnight at room temperature and visualized under 200 kV TEM (Tecnai G20 TWIN, FEI, USA).

### Statistical analyses

All data were presented as mean ± standard deviation (SD). Significant differences between two groups were determined using unpaired Student’s two tailed *t*-test. Comparisons of more than two groups were analyzed using One-way ANOVA with Bonferroni post hoc test. Statistical analysis was performed using SPSS 25.0 software. *P* < 0.05 was considered statistically significant.

## Results

### Characterization of rat EphB2-transfected BMSCs and EVs

**Fig. 1:**
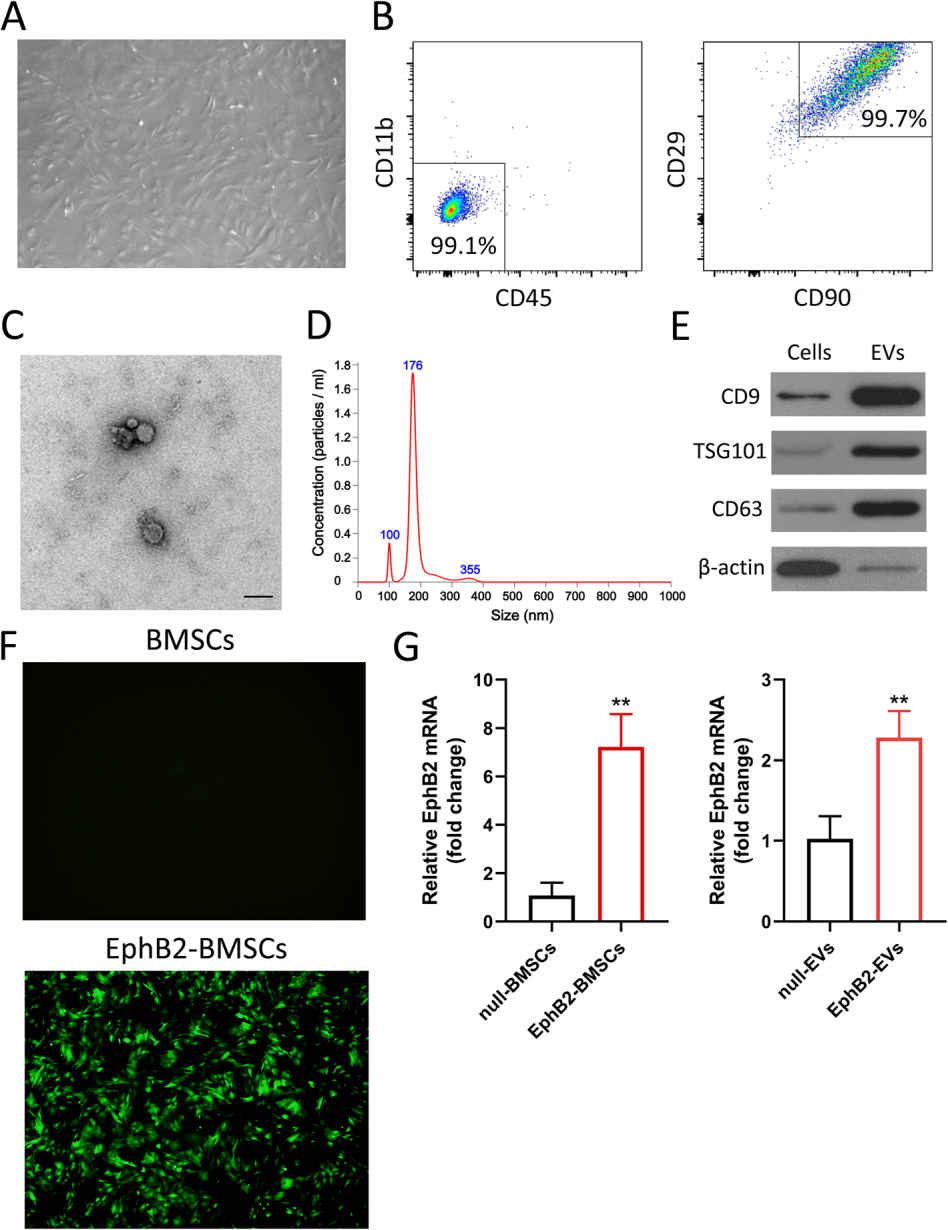
Characterization of rat EphB2-transfected BMSCs and EVs. **a** Representative image of Passage 3 (P3) BMSCs. Original magnification, × 100. **b** Flow cytometry characterization of P3 BMSCs as CD45^−^ CD11b^−^ CD90^+^ CD29^+^. **c** Transmission electron microscopy (TEM) analysis of EVs derived from BMSCs. Scale bar, 200 nm. **d** The size distribution profile of the EVs by NanoSight. **e** Western blotting analysis of EVs specific marker proteins CD9, TSG101, and CD63 in BMSCs and BMSCs-derived EVs. **f** Fluorescence microscope imaging of null-BMSCs and GFP-labeled EphB2-BMSCs. Original magnification, × 100. **g** qRT-PCR analysis of EphB2 expression in the indicated cells (left) or EVs (right). The expression level of the mRNA was normalized to β-actin. **P < 0.01 vs. DSS group


The BMSCs had the property of plastic adhesion and distributed as swirling under the microscope (Fig. 1a). Flow cytometry analysis revealed that the BMSCs were highly positive for both CD29 and CD90 (99.7%) but negative for CD11b and CD45 (99.1%) (Fig. 1b, Figure S2). Isolated EVs were characterized using different methods including TEM, NTA, and WB. EVs showed the typically spherically shape with a size range of 100–200 nm as measured by TEM (Fig. 1c). NTA confirmed the average diameter of EVs was 183.5 nm, in line with the TEM results (Fig. 1d). The EVs were further characterized using WB, showing high level expression of EVs-specific markers, including TSG101, CD63, and CD9 (Fig. 1e). The NTA video was presented in Video S1.

**Additional file 5: Video S1.** The NTA video of EVs.

To establish a stable cell line over-expressing EphB2, lentiviral vector was employed. Fluorescence labeling and qRT-PCR analysis were performed to detect EphB2 expression. Seventy-two hours after lentivirus transduction with a multiplicity of infection of 35, gcGFP expression was observed in the transfected BMSCs under a fluorescence microscope (Fig. 1f). The expression of EphB2 in the EphB2-BMSCs was 6.69-fold higher than that in the negative control cells (*P* < 0.01) (Fig. 1g). Moreover, the level of EphB2 in EphB2-EVs was 2.24-fold higher than that in the null-EVs (*P* < 0.01) (Fig. 1g). Taken together, these results indicated that lentivirus-mediated over-expression system effectively induced EphB2 over-expression in BMSCs and corresponding EphB2-EVs were acquired successfully for following studies.

### EphB2/ephrin-B1 axis promotes the homing of EVs to inflammatory colon tissue

**Fig. 2:**
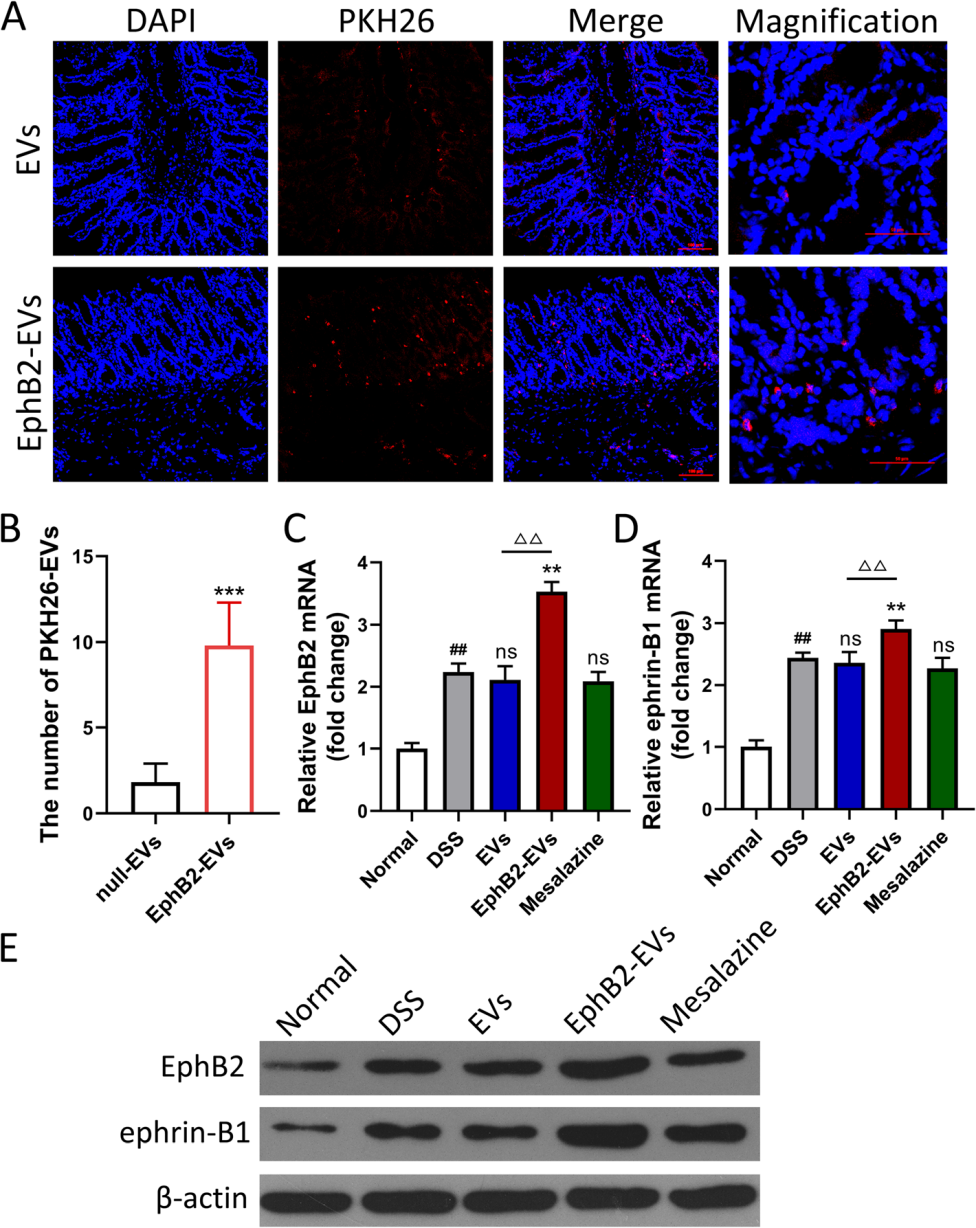
EphB2/ephrin-B1 axis promotes the homing of EVs to inflammatory colon tissue. **a** Confocal microscopy analysis of the localization of PKH26-labeled EVs and EphB2-EVs (red) in inflamed colon tissues. The cell nuclei were stained with DAPI (blue). Scale bar, 100 μm. Magnification images scale bar, 50 μm. **b** The number of PKH26-labeled EVs in each microscopic field was counted (magnification × 400), and 5 horizons were selected for each sample. qRT-PCR analysis of EphB2 (**c**) and ephrin-B1 (**d**) expression in colon tissues with indicated treatments. The expression level of the mRNA was normalized to β-actin. **e** Western blotting analysis of EphB2 and ephrin-B1 expressions in colon tissue. β-Actin was used as a loading control. ***P < 0.001 vs. null-EVs group, ^##^*P* < 0.01 vs. normal group, **P < 0.01 vs. DSS group, ^△△^*P* < 0.01 vs. EVs group

To track the location and distribution of EVs and EphB2-EVs in the inflamed colon, same dosage of PKH26-lableded EVs and EphB2-EVs were injected into colitis rats via the tail vein. Rats were sacrificed 12 h after injection, and colon tissues were collected and prepared to make frozen section. The tissue staining showed that the PKH26-labeled EVs and EphB2-EVs were confirmed as red dots (Fig. 2a). While both EVs and EphB2-EVs could indeed migrate to the damaged colon tissue, a significantly higher proportion of EphB2-EVs were observed in the lesion area compared to that of EVs (Fig. 2a, b). It is a critical step in amplifying biological effect of EVs. Consistent with this result, a marked increase in the expression levels of EphB2 and ephrin-B1 were observed in the damaged colons of colitis rats administrated with EphB2-EVs compared to the rats administrated with EVs. What is more, the ephrin-B1 was in a positive correlation with EphB2 (Fig. 2c–e). These results revealed that EphB2/ephrin-B1 axis enhances the migration efficiency of EVs towards the lesion colon.

### Effect of EphB2-EVs on disease progression of DSS colitis rats

**Fig. 3:**
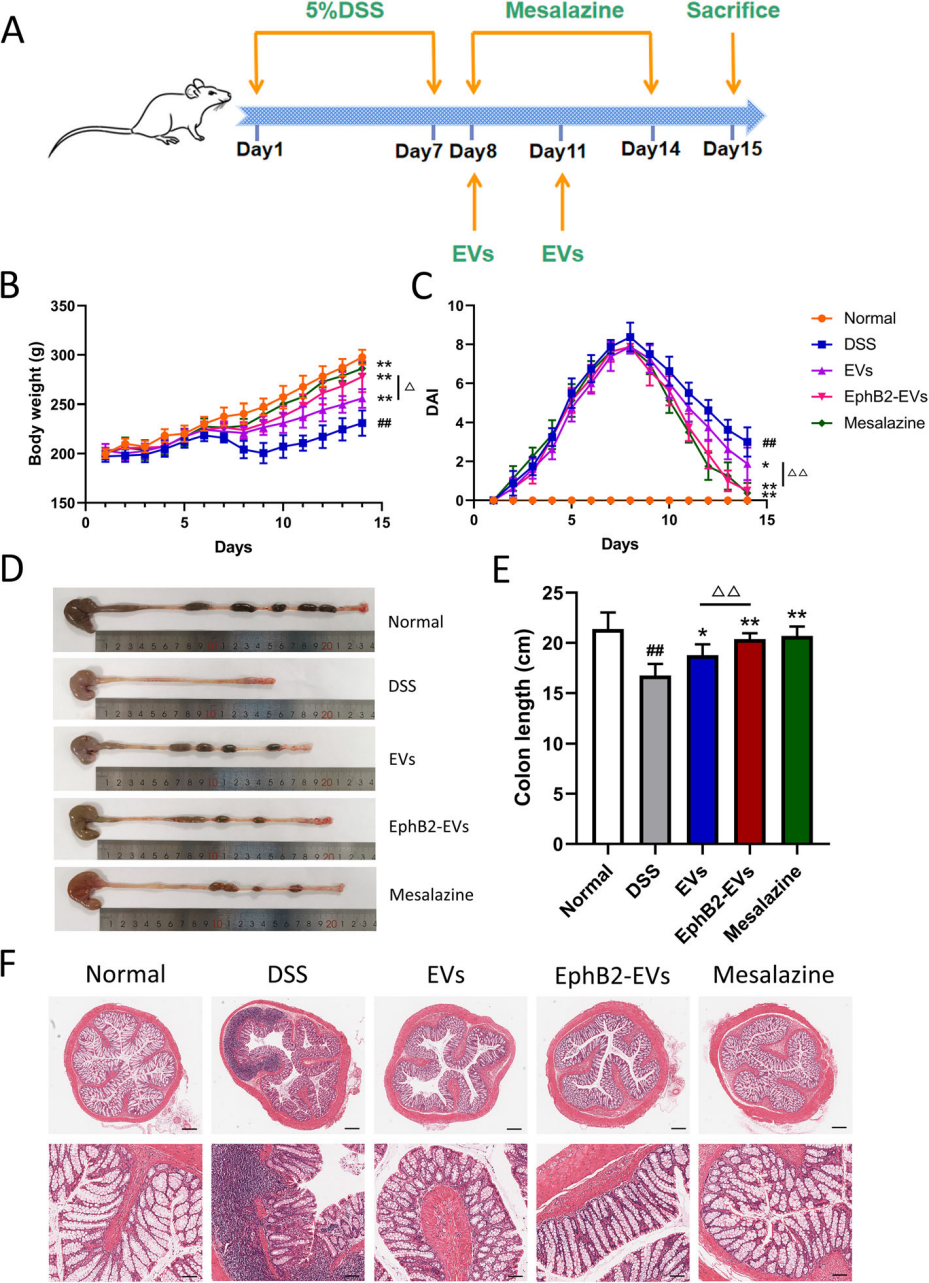
EphB2-EVs ameliorate DSS-induced colitis. **a** Experiment time-points for treatments. **b** Changes in body weight. **c** DAI score of rats. Representative picture (**d**) and length quantification (**e**) of colon from each group. **f** Histopathological changes in colon tissues analyzed by hematoxylin and eosin (HE) staining. Scale bar, 400 μm (upper) and 100 μm (lower). ^##^*P* < 0.01 vs. normal group; *P < 0.05, **P < 0.01 vs. DSS group; ^△△^*P* < 0.01 vs. EVs group

To investigate the therapeutic effect of EphB2-EVs in inflammatory bowel disease, DSS-induced rat colitis model was used (Fig. 3a). The DAI scores were recorded daily to estimate the severity of symptoms, such as body weights, stool consistency, and stool occult blood. At day 5 after DSS administration, a significant increase in the DAI score was observed in all of the model rats, manifested by severe diarrhea, bloody stools, and slow weight gain (Fig. 3b, c). However, compared to the DSS group, animals treated with EphB2-EVs exhibited significantly reduced DAI score (Fig. 3c). In addition, the shortening of colon length, a key characteristic of damaged inflammatory colon, was markedly reversed in the EphB2-EVs group and mesalazine group (positive control) compared to the DSS group (Fig. 3d, e). The disease severity of colitis was further evaluated by histopathological analysis. In the DSS group, colon tissue demonstrated obvious structure disorder, mucosal ulceration, epithelial damage, gland swelling, and inflammatory cell infiltration. Moreover, a significantly reduced histological damage was observed in both EphB2-EVs and mesalazine groups (Fig. 3f). These results demonstrate that EphB2-EVs effectively ameliorate the intestinal injury in DSS-induced colitis rats.

### EphB2-EVs attenuate DSS-induced inflammation and oxidative stress

**Fig. 4:**
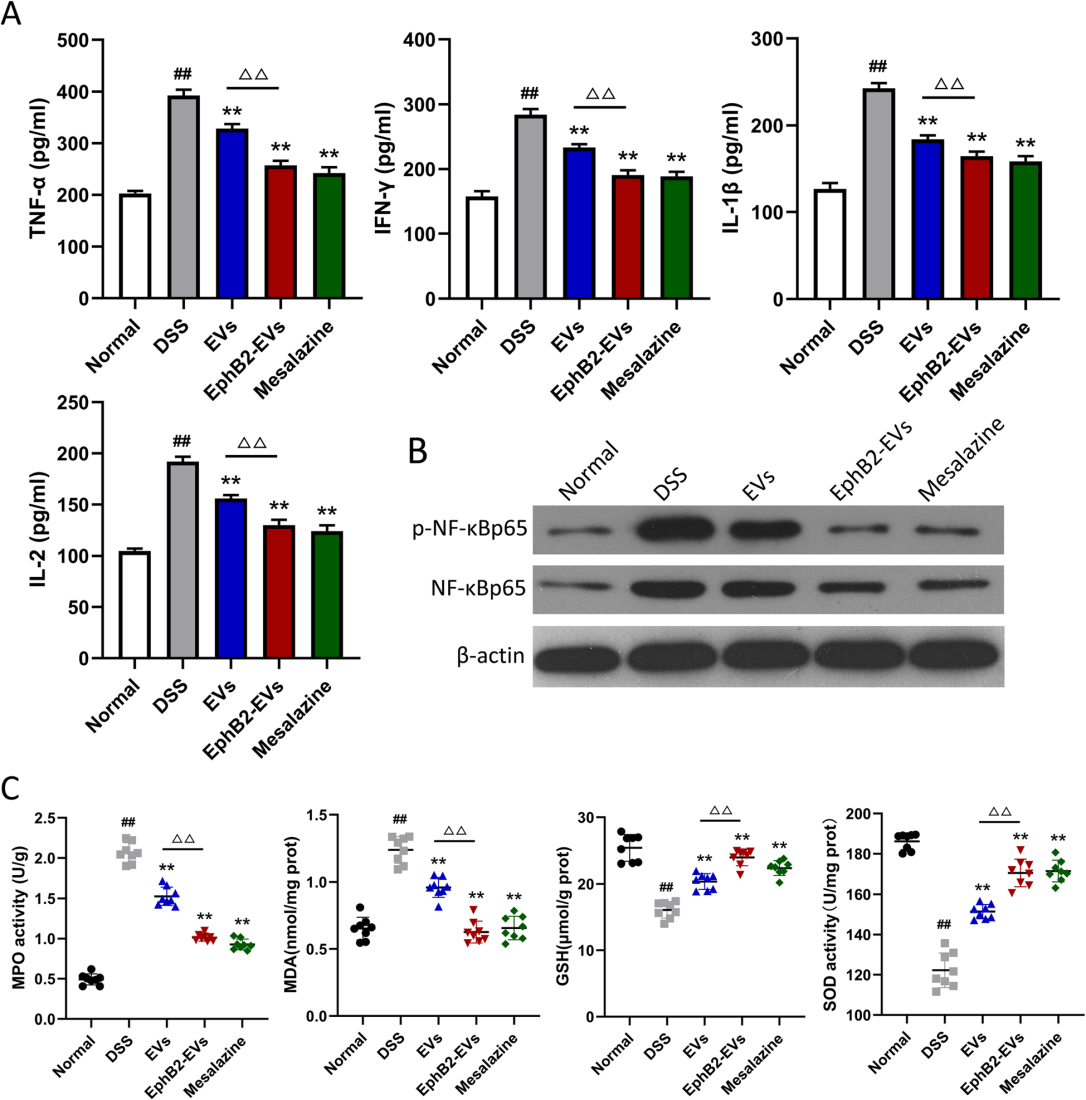
EphB2-EVs suppress the production of inflammatory mediators and alleviate DSS-induced oxidative stress in colitis rats. **a** Protein levels of TNF-α, IFN-γ, IL-1β, and IL-2 in colon tissues homogenates from indicated treatment groups by ELISA. **b** Western blotting analysis of NF-κBp65 and p-NF-κBp65. β-Actin was used as a loading control. **c** Expression levels of MPO, MDA, GSH, and SOD in colon tissues from indicated treatment groups. ^##^*P* < 0.01 vs. normal group, **P < 0.01 vs. DSS group, ^△△^*P* < 0.01 vs. EVs group

To further evaluate the effect of EphB2-EVs on the inflammatory response, the levels of inflammatory mediators were measured in the colon tissues. As shown in Fig. 4a, the protein expression of the proinflammatory cytokines, including TNF-α, IFN-γ, IL-1β, and IL-2, were much lower in EphB2-EVs group and mesalazine group than those in the DSS group. The transcription factor NF-κB plays a key role in the expression of the above cytokines. Consistent with the above findings, a significant decrease in the expression levels of both NF-κB p65 and phospho-NF-κB p65 was observed in the EphB2-EVs and mesalazine groups compared to the DSS group (Fig. 4b). MPO, MDA, GSH, and SOD are known to be involved in oxidative stress and inflammatory response. Here, we evaluated the status of oxidative stress in colitis colon with different treatment. Animals treated with DSS exhibited increased activity of MPO, elevated MDA, and decreased SOD and GSH (Fig. 4c). Compared to the DSS group, the EphB2-EVs and mesalazine groups exhibited much less abnormality in the levels of MPO, MDA, GSH, and SOD (Fig. 4c). These findings manifest that EphB2-EVs attenuate DSS-induced oxidative stress and inflammation in colon tissue.

### EphB2-EVs protect the intestinal epithelial barrier function in DSS colitis rats

**Fig. 5:**
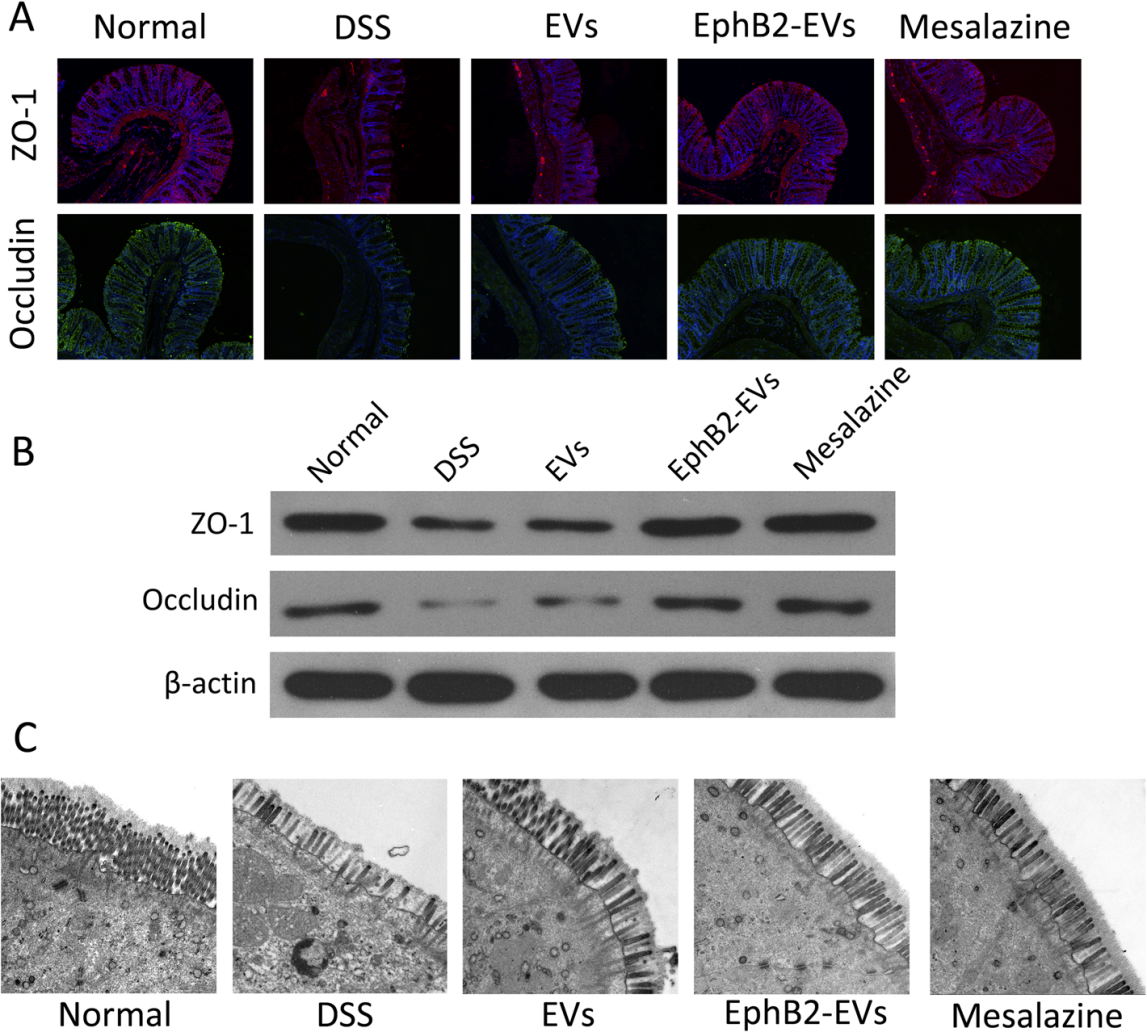
EphB2-EVs protect intestinal epithelial barrier from disruption. **a** The expressions of tight junction associated proteins ZO-1 (red) and occludin (green) in colon tissue were examined with immunofluorescence staining, and the nuclei were stained with DAPI. Original magnification, × 100. **b** The expression of ZO-1 and occludin detected by western blotting, and β-actin was used as a loading control. **c** The ultrastructure of the colonic mucosal tissues of rats were examined by transmission electron microscopy (TEM). Original magnification, × 5000

Tight junctions (TJs) of intestinal epithelium are the essential barrier against exogenous damage. The interaction between ZO-1 and occludin plays a pivotal role in maintaining epithelial barrier integrity. To explore the effects of EphB2-EVs on the epithelial reconstruction, we assessed the expression of ZO-1 and occludin by means of immunofluorescence and WB. Compared to the DSS group showing obvious decline of ZO-1 and occludin, EphB2-EVs- and mesalazine-treated groups exhibited significantly less damage of the intestinal epithelial integrity (Fig. 5a, b). These results were further confirmed by the ultrastructure observation using TEM, which showed that TJs were broken with organelles swollen, and disordered intestinal microvilli in the colonic mucosal tissues of DSS group. In contrast, a significantly less damage of the colon ultrastructure was observed in the EphB2-EVs group (Fig. 5c). Altogether, EphB2-EVs robustly mitigate colon tissues injury and TJs destruction in DSS-induced colitis rats.

### EphB2-EVs regulate the balance of Th17/Treg cell in colitis rats

**Fig. 6:**
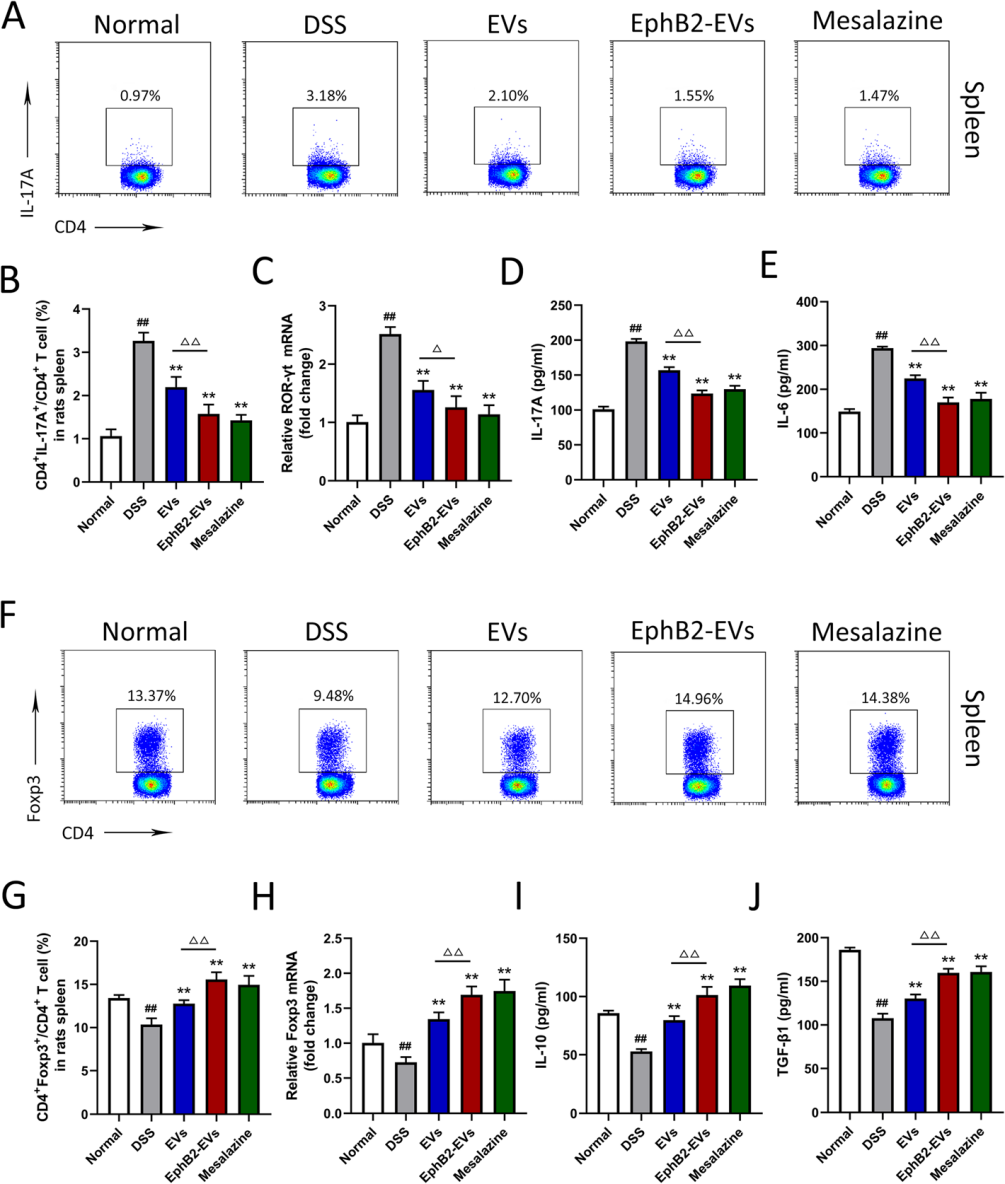
EphB2-EVs regulate the balance of Th17 and Treg cells. The representative FACS plots (**a**) and the percentages (**b**) of CD4^+^ IL-17^+^ Th17 cells in spleen were analyzed by flow cytometry. **c** qRT-PCR analysis of transcription factors ROR-γt mRNA expression in colons. The expression level of the mRNA was normalized to β-actin. ELISA analysis of IL-17A (**d**) and IL-6 (**e**) levels in colons. The representative FACS plots (**f**) and the percentages (**g**) of CD4^+^ Foxp3^+^ Treg cells in spleen were analyzed by flow cytometry. **h** qRT-PCR analysis of transcription factors Foxp3 mRNA expression in colons. The expression level of the mRNA was normalized to β-actin. ELISA analysis of IL-10 (**i**) and TGF-β1 (**j**) levels in colons. ^##^*P* < 0.01 vs. normal group, **P < 0.01 vs. DSS group, ^△△^*P* < 0.01 vs. EVs group

To explore the immunological mechanism by which EphB2-EVs modulate colon inflammation, we investigated the effects of EphB2-EVs on Th17 and Treg cells differentiation in vivo. DSS treatment resulted in a marked elevation of CD4^+^ IL-17A^+^ Th17 cells and a marked downregulation of CD4^+^Foxp3^+^ Treg cells in the spleen and MLNs (Fig. 6a, b, f, g, Figure S3a-d). Treatment with EVs, EphB2-EVs, or mesalazine resulted in significant decrease in the proportion of Th17 cells and dramatic increase in the frequency of Treg cells (Fig. 6a, b, f, g, Figure S3a-d). More significant reversal of Th17 and Treg was observed in the EphB2-EVs group compared to the EVs group (Fig. 6a, b, f, g, Figure S3a-d).

Furthermore, the Th17 and Treg related transcription factors, ROR-γt and Foxp3, respectively, as well as signature cytokines, IL-17A, IL-10, IL-6, and TGF-β1 in colon tissue were analyzed using qRT-PCR and ELISA. The mRNA expression of the ROR-γt increased, while Foxp3 slightly decreased in DSS group. Treatment with EphB2-EVs or mesalazine significantly reversed the mRNA expression of the transcription factors (Fig. 6c, h). Consistently, DSS-induced colitis rats exhibited upregulation of IL-17A and IL-6 and downregulation of IL-10 and TGF-β1 in the colonic tissues. Treatment with EphB2-EVs or mesalazine significantly reversed the changes of the above cytokines (Fig. 6d, e, i, j). These results indicated that EphB2-EVs modulate the Th17/Treg cell balance to maintain colonic immune homeostasis.

### EphB2-EVs restrain the activation of JAK-STAT3 signaling pathways in colon tissue

**Fig. 7:**
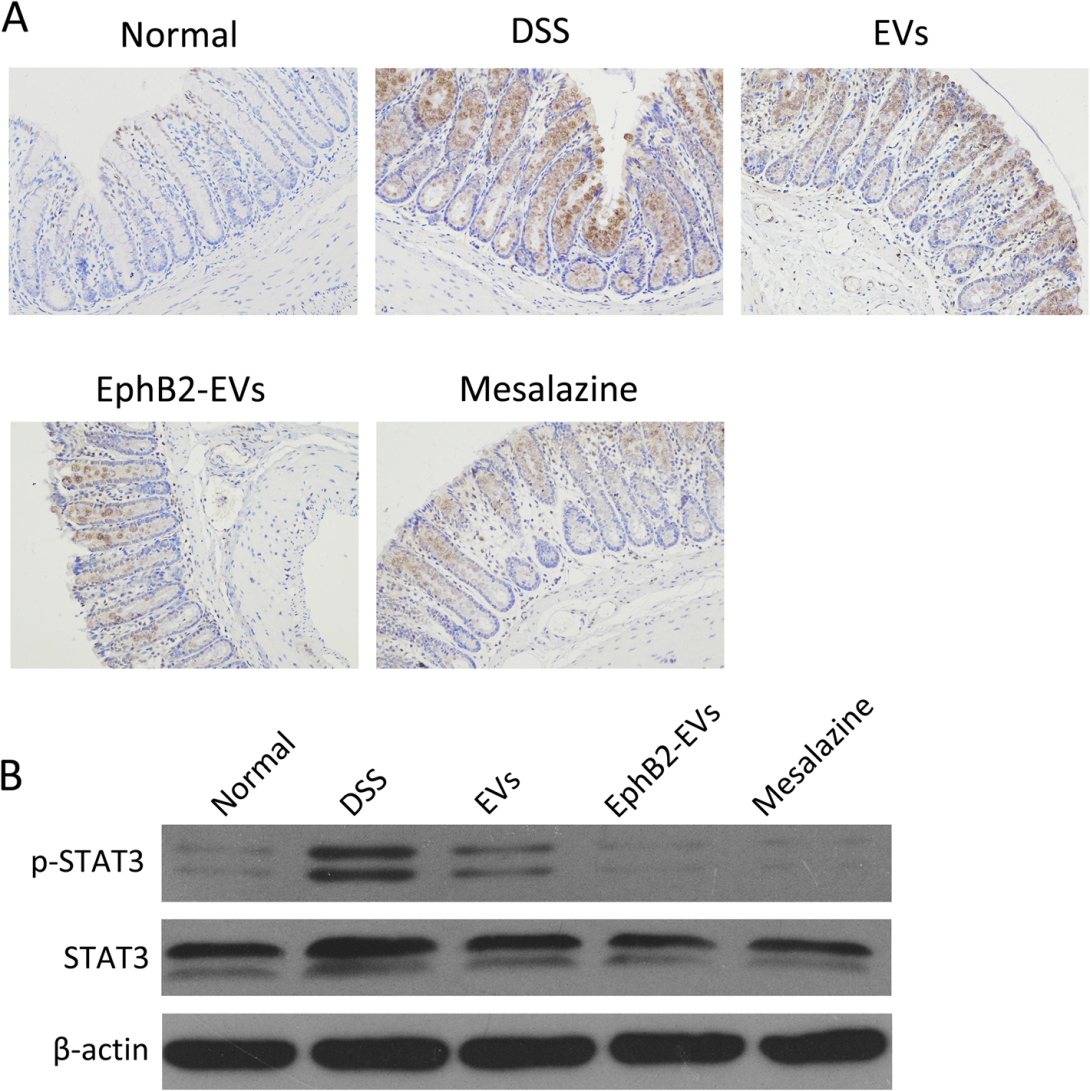
EphB2-EVs prevent the activation of JAK-STAT3 pathway in colon tissue. **a** Immunohistochemical staining for STAT3 in colon tissue. Original magnification, × 200. **b** Western blotting analysis of STAT3 and p-STAT3 in colon tissue. β-Actin was used as a loading control

The JAK-STAT pathway participates in the modulation of Th17 and Treg cells. Therefore, this signaling pathway was also investigated to further ascertain the immunomodulatory mechanism of EphB2-EVs during colitis. Strikingly, a significant downregulation of STAT3 was observed in the colon tissues of the EphB2-EVs group compared to that of the DSS group as measured by IHC (Fig. 7a). WB analysis further confirmed the downregulation of both STAT3 and p-STAT3 in the colon tissues of the EphB2-EVs group compared with the DSS group (Fig. 7b). These data suggest that EphB2-EVs-mediated inhibition of STAT3 may play a role in restoring the immune homeostasis.

## Discussion

Although the precise pathogenesis of UC remains undefined, inflammation and intestinal mucosal immune disorder are considered to be the critical initiation factors for UC [46, 47]. Current available therapeutic medicines for UC have many limitations, including the side effects and high rate of drug resistance [5]. Therefore, optimal alternative therapeutic strategies are imminently needed. In the present study, we systematically studied the therapeutic effects of EphB2-EVs on DSS-induced colitis rats and its potential mechanism (Figure S4). To our knowledge, this is the first study showing efficacy of EphB2-EVs in colitis animal model and paves the way for the clinical application of EphB2-EVs to UC patients.

It was reported that the expression of eprin-B1, the ligand of EphB2, was elevated in the inflammatory intestine, and EphB2/ephrin-B1 axis regulates cell migration [16, 48]. We investigated whether EphB2 over-expressed EVs could enhance the homing ability of EVs to damaged colon tissue through EphB2/ephrin-B1 axis. We observed that both EVs and EphB2-EVs could migrate to the damaged intestinal tissue after tail vein injection, and EphB2-EVs showed super homing efficiency to targeted tissue. Additionally, the accumulation of EVs in the colon tissue was positively correlated with the presence of the EphB2. Mechanically, EphB2/ephrin-B1 axis induced migration at least partly enhanced the targeting efficiency. Our study strongly suggested that EphB2-EVs could abundantly migrate to the damaged intestinal tissues.

EphB2 plays a role in repairing tissue damage and regulating inflammatory response [18, 49]. Our previous studies have shown that BMSC-EVs ameliorate inflammation in the colon [35, 37]. Therefore, we speculated that EphB2-EVs may be able to relieve DSS-induced colitis in rats. The DSS-induced colitis model is widely used in preclinical studies [50]. In our research, DSS-induced colitis models successfully mimicked the clinical symptoms of UC, including body weight loss, bloody stools, diarrhea, and colon shortening. Compared with EVs, EphB2-EVs significantly ameliorated the clinical symptoms of colitis in rats, accompanied by the amelioration of inflammatory response in the colon tissue. In DSS-induced colitis rats, the inflamed intestine can produce abundant abnormal inflammatory mediators. We further demonstrated that EphB2-EVs significantly inhibited the activation of NF-κB, reduced the level of pro-inflammatory cytokines, such as TNF-α, IFN-γ, IL-1β, and IL-2, and upregulated the anti-inflammatory cytokines, including IL-10 and TGF-β1, in the inflamed colon tissue. Thus, EphB2-EVs are effective to attenuate inflammation in intestinal mucosa and restore the damaged colon tissue.

Oxidative stress, as important as inflammatory response, is involved in the pathological process of inflammatory bowel disease [51]. On the one hand, massive inflammatory factors release disrupted the balance of oxidants and antioxidants [51]. On the other hand, the occurrence of oxidative stress further exacerbates the inflammatory response [52, 53]. As expected, we observed that after treatment with EphB2-EVs, the MPO activity and MDA expression were significantly decreased in DSS-induced colitis, while the activity of SOD and GSH level were remarkably increased. These data indicate that EphB2-EVs effectively reduce the oxidative stress.

With the aggravation of abnormal oxidative stress and inflammation, the intestinal mucosal barrier integrity was disrupted, resulting in the exacerbation of UC [51]. The intestinal epithelial as the fundamental barrier plays an essential role in protecting homeostasis against exogenous antigens and damage. ZO-1 and Occludin were two major tight junctions (TJ) proteins in intestine [54]. In the inflamed colon tissue, we observed large absence of ZO-1 and occludin in DSS-induced colitis rats. Moreover, Eph/ephrin signaling plays an important role in cell-cell junctions to maintain tissue homeostasis. Our study indicated that EphB2-EVs treatment significantly elevated the expression of ZO-1 and occludin in the damaged colon. Furthermore, we showed that EphB2-EVs reversed the damaged intestinal mucosa ultrastructure. Hence, EphB2-EVs are beneficial to maintaining intestinal mucosal barrier function and promoting the repair of damaged intestinal tissues. All these data suggest that EphB2-EVs could inhibit the release of inflammatory mediators and attenuate oxidative stress, thereby repairing the intestinal mucosal barrier in the UC rats.

Imbalance of Th17 and Treg cells is directly involved in the occurrence of UC [55]. EphB/ephrinB signaling has a potential role in regulating the function of immune cells. Previous studies have found that EphB2/ephrin-B1 interaction is critical for MSC-mediated suppression of T cell activation and proliferation. This effect is mediated mainly through ephrin-B1 reverse signaling [21]. Herein, we further explored the effect of EphB2-EVs on Th17/Treg cells balance. Our research showed that along with the aggregation of Th17 cells, the Treg cells showed a relative absence in the colitis rats. EphB2-EVs treatment remarkably reduced Th17 cells, and upregulated Treg cells in spleen and MLNs. These were associated with dramatic decrease in Th17-related ROR-γt and IL-17A, and increase in the Treg-related Foxp3 and IL-10 expression. Thus, EphB2-EVs were beneficial to maintaining the balance of Th17/Treg cells in both colon tissue and immune organ.

The differentiation towards Th17 or Treg cells predominantly depends on the concentrations of IL-6 and TGF-β1 and the activation of STAT3. Previous reports have shown that the naive T cells exposed to low concentration of TGF-β1 along with plentiful IL-6 could drive the differentiation towards Th17 cells, and high concentration of TGF-β1 are able to modulate the differentiation of Treg cells [56].

Consistent with these findings, our research revealed that after treatment with EphB2-EVs, IL-6 was remarkably downregulated, whereas TGF-β1 was significantly increased. Moreover, we observed that EphB2-EVs inhibited the phosphorylation of STAT3 in the colon tissue. Based on these data, we conclude that EphB2-EVs could regulate Th17 and Treg cells differentiation via modulating the expression of IL-6 and TGF-β1 and inhibiting the activation of STAT3. Our current study provides new information as to the mechanism by which EphB2-EVs ameliorate colonic tissue damage and at the same time points the way towards a potentially highly effective form of EV-based therapy for UC.

## Conclusion

In summary, the presented study demonstrated that over-expression of EphB2 on EVs enhanced the enrichment of EVs in the damaged colon tissues. Compared to EVs, EphB2-EVs remarkably restored the intestinal mucosal barrier and attenuated the inflammatory response and oxidative stress in DSS-induced colitis rats. Furthermore, EphB2-EVs hold a robust immunomodulatory effect on the Th17/Treg balance by inhibiting JAK-STAT pathway and regulating the secretion of IL-6 and TGF-β1. Thus, our study indicate that EphB2-EVs function as a potential approach for UC treatment, although the precise molecular mechanism by which EphB2-EVs inhibit JAK/STAT signaling pathways remains to be investigated.

## Supplementary Information

**Additional file 1: Figure S1.** Experimental outline for the extraction process of EphB2-EVs using ultracentrifugation.

**Additional file 2: Figure S2.** The percentages of different BMSCs markers.

**Additional file 3: Figure S3.** The percentages of Th17 cells and Treg cells in MLNs. (A and C) The representative FACS plots (A) and the percentages (C) of CD4+ IL-17+ Th17 cells in MLNs were analyzed by flow cytometry. (B and D) The representative FACS plots (B) and the percentages (D) of CD4+ Foxp3+ Treg cells in MLNs were analyzed by flow cytometry.

**Additional file 4: Figure S4.** Schematic depiction about the therapeutic effects of EphB2-EVs.

**Additional file 6: Table S1.** Scoring of disease activity index (DAI).

**Additional file 7: Table S2.** Sequences of primers for qRT-PCR analysis.

## Data Availability

The datasets used and/or analyzed during the current study are available from the corresponding author on reasonable request.

## Abbreviations

BMSCs: Bone mesenchymal stem cells
EVs: Extracellular vesicles
UC: Ulcerative colitis
EphB2: Ephrin type B receptor 2
RTKs: Receptor tyrosine kinases
DAI: Disease activity index
DSS: Dextran sulfate sodium
IBD: Inflammatory bowel disease
MPO: Myeloperoxidase
MDA: Malondialdehyde
SOD: Superoxide dismutase
GSH: Glutathione
TJs: Tight junctions
MLNs: Mesenteric lymph nodes
Th17 cells: T helper 17 cells
Treg cells: Regulatory T cells
