# Supplementary figures and images for: Extracellular vesicles derived from EphB2-overexpressing bone marrow mesenchymal stem cells ameliorate DSS-induced colitis by modulating immune balance
Source: Stem Cell Res Ther. 2021 Mar 15;12:181. doi: 10.1186/s13287-021-02232-w (PMC7962309; doi:10.1186/s13287-021-02232-w)

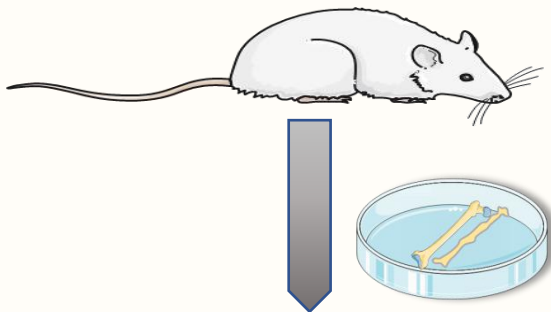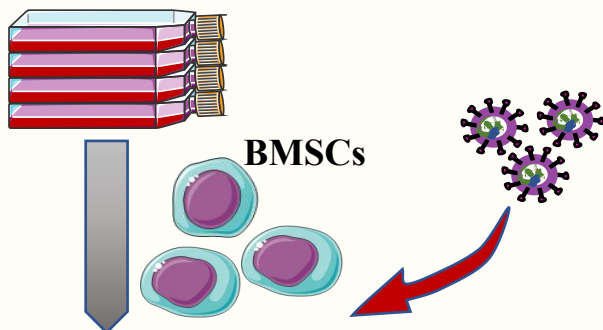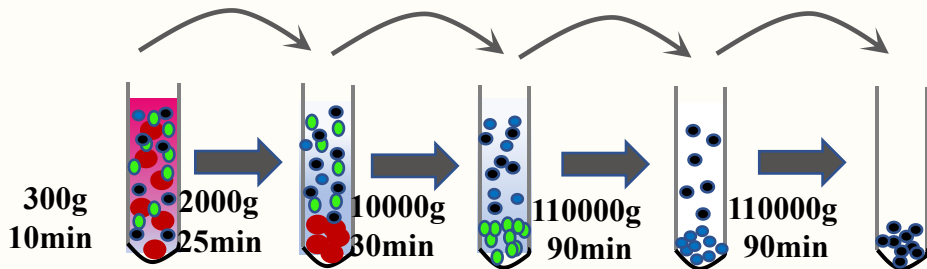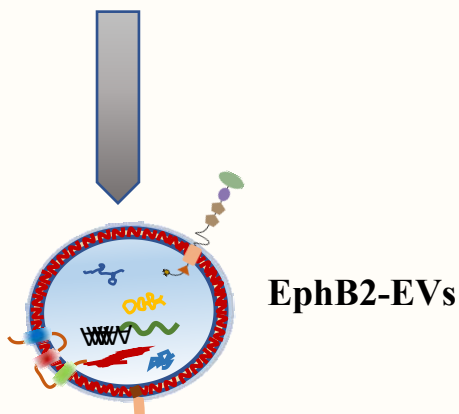

Supplement: Supplementary file 2 — Additional file 1: Figure S1. Experimental outline for the extraction process of EphB2-EVs using ultracentrifugation. [file 13287_2021_2232_MOESM1_ESM.pdf]

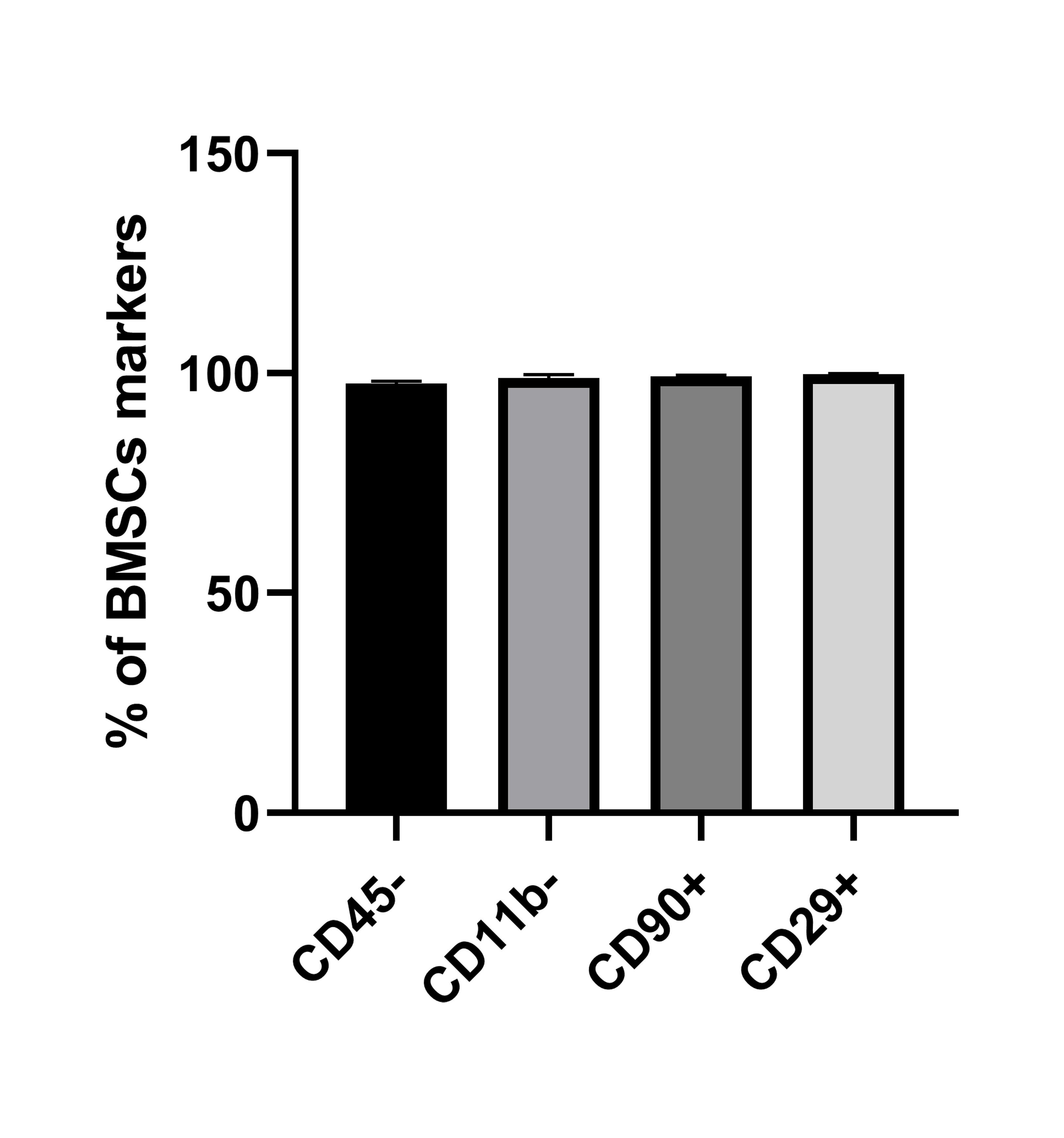

Supplement: Supplementary file 3 — Additional file 2: Figure S2. The percentages of different BMSCs markers. [file 13287_2021_2232_MOESM2_ESM.tif]

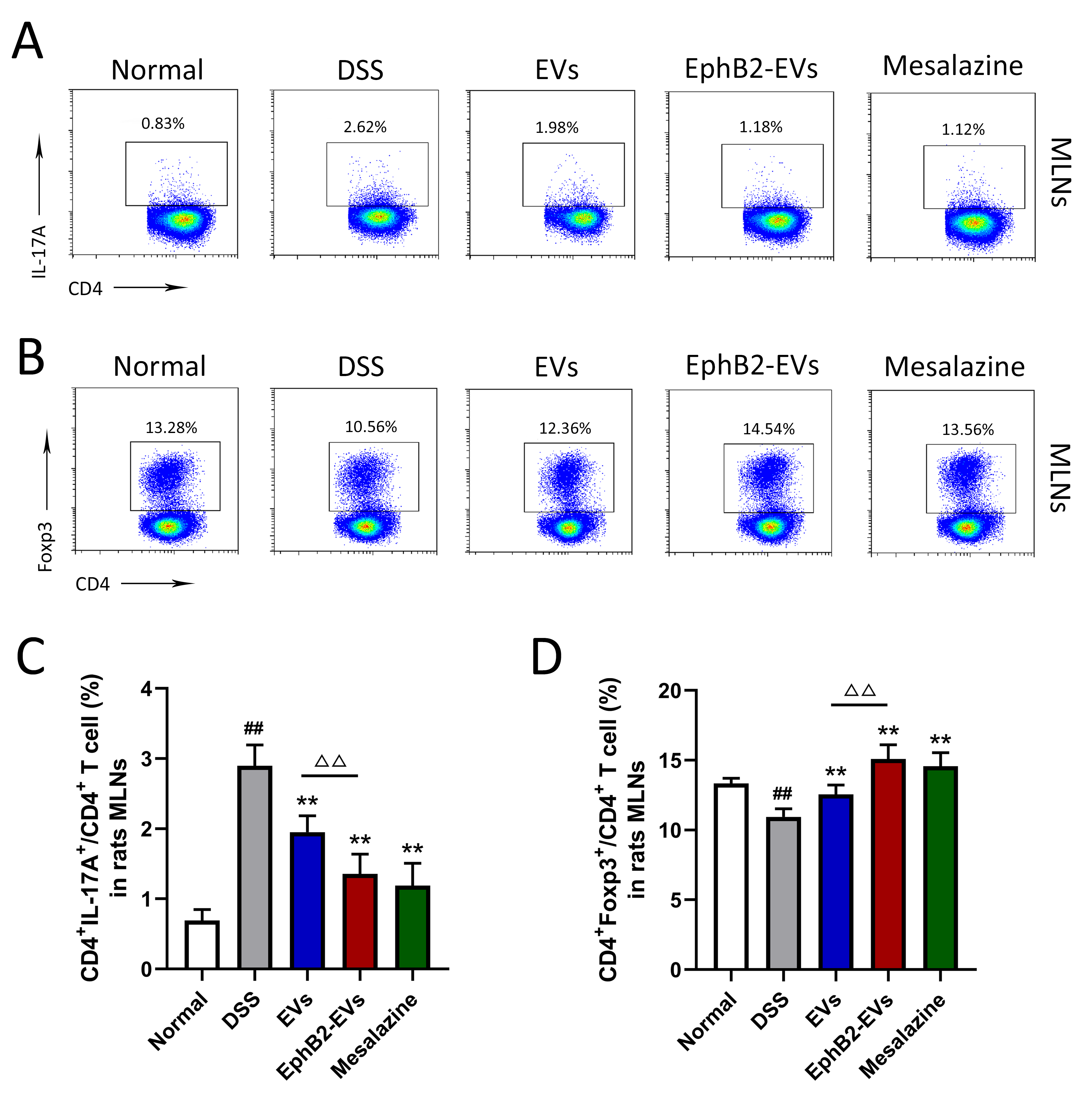

Supplement: Supplementary file 4 — Additional file 3: Figure S3. The percentages of Th17 cells and Treg cells in MLNs. (A and C) The representative FACS plots (A) and the percentages (C) of CD4+ IL-17+ Th17 cells in MLNs were analyzed by flow cytometry. (B and D) The representative FACS plots (B) and the percentages (D) of CD4+ Foxp3+ Treg cells in MLNs were analyzed by flow cytometry. [file 13287_2021_2232_MOESM3_ESM.tif]
