# Supplementary material for: Extracellular vesicles derived from EphB2-overexpressing bone marrow mesenchymal stem cells ameliorate DSS-induced colitis by modulating immune balance
Source: Stem Cell Res Ther. 2021 Mar 15;12:181. doi: 10.1186/s13287-021-02232-w (PMC7962309; doi:10.1186/s13287-021-02232-w)
Supplement: Supplementary file 5 — Additional file 4: Figure S4. Schematic depiction about the therapeutic effects of EphB2-EVs. [file 13287_2021_2232_MOESM4_ESM.pdf]

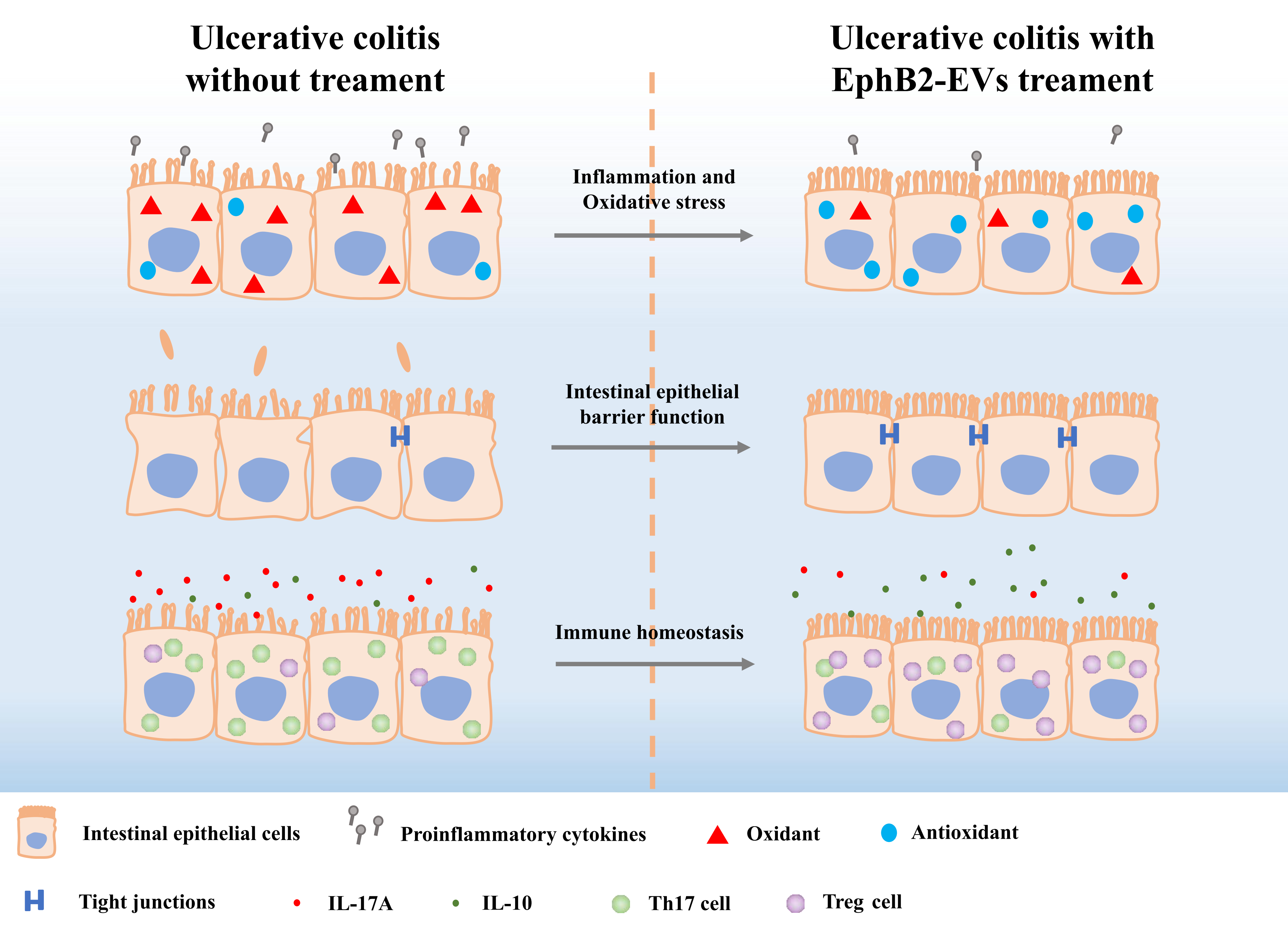
Ulcerative colitis without treament
Ulcerative colitis with EphB2-EVs treament
Inflammation and Oxidative stress
Intestinal epithelial barrier function
Immune homeostasis
Intestinal epithelial cells
Proinflammatory cytokines
Oxidant
Antioxidant
Tight junctions
IL-17A
IL-10
Th17 cell
Treg cell
