# Supplementary material for: Extracellular vesicles derived from EphB2-overexpressing bone marrow mesenchymal stem cells ameliorate DSS-induced colitis by modulating immune balance
Source: Stem Cell Res Ther. 2021 Mar 15;12:181. doi: 10.1186/s13287-021-02232-w (PMC7962309; doi:10.1186/s13287-021-02232-w)
Supplement: Supplementary file 6 — Additional file 6: Table S1. Scoring of disease activity index (DAI). [file 13287_2021_2232_MOESM6_ESM.pdf]

Score	Weight loss	Stool consistency	Occult blood
0	<1%	Normal	Negative
1	1–5%	Soft but formed	Weakly positive
2	5–10%	Soft	Positive
3	10–15%	Soft and wet	Strongly positive
4	>15%	Liquid	Visible
