# Supplementary material for: Extracellular vesicles derived from EphB2-overexpressing bone marrow mesenchymal stem cells ameliorate DSS-induced colitis by modulating immune balance
Source: Stem Cell Res Ther. 2021 Mar 15;12:181. doi: 10.1186/s13287-021-02232-w (PMC7962309; doi:10.1186/s13287-021-02232-w)
Supplement: Supplementary file 7 — Additional file 7: Table S2. Sequences of primers for qRT-PCR analysis. [file 13287_2021_2232_MOESM7_ESM.pdf]

Gene name		Primer sequences (5′ to 3′)
EphB2	Forward	TTATGGCTGTGGCGTTGT
	Reverse	CATCACCCTGTCGTGGTC
ephrin-B1	Forward	TTACACCACCCACCTCCAC
	Reverse	CCTTCCGACTGCCACCT
ROR-γt	Forward	ACCCACACCTCACAAATCG
	Reverse	GGAGTAGGCCACATTGCAC
Foxp3	Forward	AGTACCCCCAAATTCCTGCCT
	Reverse	CCACTGTTAGGGTATGGGAGG
β-actin	Forward	CCCGCGAGTACAACCTTCTTG
	Reverse	CTGACCCATACCCACCATCAC
